# Impact of Sex-Specific Preoperative Fat Mass Assessment on Long-Term Prognosis after Gastrectomy for Gastric Cancer

**DOI:** 10.3390/cancers15072100

**Published:** 2023-03-31

**Authors:** Ryota Matsui, Noriyuki Inaki, Toshikatsu Tsuji, Tetsu Fukunaga

**Affiliations:** 1 Department of Gastroenterological Surgery, Ishikawa Prefectural Central Hospital, Kanazawa 920-8530, Japan; 2 Department of Upper Gastrointestinal Surgery, Juntendo University Hospital, Tokyo 113-8431, Japan; 3 Department of Gastrointestinal Surgery/Breast Surgery, Graduate School of Medical Science, Kanazawa University, Kanazawa 920-8641, Japan; 4 Department of Gastroenterological Surgery, The Cancer Institute Hospital of Japanese Foundation for Cancer Research, Tokyo 135-8550, Japan

**Keywords:** gastric cancer, obesity paradox, overall survival, subcutaneous fat, visceral fat

## Abstract

**Simple Summary:**

Gastric cancer has a different characteristic than other cancers in that it causes postoperative body weight loss associated with gastric volume loss. It has been reported that the greater the rate of body weight loss after gastrectomy, the poorer the long-term prognosis. Therefore, a higher preoperative fat content may be a nutritional advantage. However, it is controversial whether a higher fat mass is associated with a better prognosis. In obesity, men tend to have visceral fat and women tend to have subcutaneous fat. In order to clarify the prognostic impact of obesity, it is necessary to investigate the prognostic relevance of fat distribution by sex. This study revealed that a low visceral fat mass in men and a low subcutaneous fat mass in women were independent poor prognostic factors after radical gastrectomy for advanced gastric cancer.

**Abstract:**

We investigated the impact of the difference in fat distribution between men and women on long-term prognosis after gastrectomy in patients with advanced gastric cancer. Patients with advanced gastric cancer deeper than p-T2 who underwent gastrectomy between April 2008 and June 2018 were included. Visceral fat mass index (VFI) and subcutaneous fat mass index (SFI) were calculated by dividing the cross-sectional area at the umbilical level by the height squared. The medians of VFI and SFI by sex were defined as cut-off values, below which values were defined as low VFI and low SFI. Of the 485 patients, 323 (66.6%) were men and 162 (33.4%) were women. Men with a low VFI had a significantly worse overall survival (OS) (*p* = 0.004) and women with a low SFI had a significantly worse OS (*p* = 0.007). Patients with a low VFI and low SFI had the worst prognosis. Multivariate analysis showed that a low VFI was an independent poor prognostic factor in men, while a low SFI was an independent poor prognostic factor in women. In conclusion, a low visceral fat mass in men and a low subcutaneous fat mass in women were independent poor prognostic factors after radical gastrectomy for advanced gastric cancer.

## 1. Introduction

A preoperative body composition assessment was recently reported as useful for predicting long-term prognosis. Since it has been reported that sarcopenia, which is caused by loss of skeletal muscle mass, is a poor prognostic factor [1,2,3,4], body composition assessments have been attracting attention. On the other hand, although a high visceral fat mass is reportedly a risk factor for postoperative complications [5,6,7,8,9,10,11,12], consensus is lacking about its prognostic impact. Since obesity rates are on the rise worldwide, it is important to clarify the prognostic impact of fat mass.

No studies have separately examined the relationship between prognosis and visceral and subcutaneous fat mass in men and women in advanced gastric cancer patients after gastrectomy. In obesity, men tend to have visceral fat and women tend to have subcutaneous fat. In men, a high ratio of visceral to subcutaneous fat mass is associated with poor prognosis in prostate cancer, and in women, a high visceral fat mass is a poor prognostic factor in breast cancer [13,14]. However, we reported that a low visceral fat mass is a poor prognostic factor in advanced gastric cancer patients after gastrectomy [15]. Although a high fat content reflects nutrient accumulation, differences in its distribution may affect long-term prognosis because body weight loss occurs after gastrectomy due to decreased gastric volume. Therefore, these results may affect long-term prognosis after gastrectomy differently in men and women.

This study aimed to clarify the impact of the differences in preoperative fat mass distribution between men and women on long-term prognosis after gastrectomy in advanced gastric cancer patients. We hypothesized that men with a high visceral fat mass and women with a high subcutaneous fat mass would have a better prognosis than their low-fat-mass counterparts.

## 2. Materials and Methods

### 2.1. Patients

This single-institution, retrospective cohort study was conducted at our institution and included 485 consecutive patients who underwent gastrectomy for primary p-T2 or advanced gastric cancer diagnosed between April 2008 and June 2018. The inclusion criteria were as follows: (1) diagnosis of primary gastric cancer; (2) history of gastrectomy; and (3) availability of computed tomography (CT) images with data on preoperative visceral fat area, subcutaneous fat area, and skeletal muscle mass. The exclusion criteria were as follows: (1) early gastric cancer; (2) residual gastric cancer; (3) cancers of other organs; (4) previous non-gastrectomy surgical procedures; (5) unresectable distant metastases; (6) received preoperative treatment; and (7) those with insufficient CT image data. We included patients who had received adjuvant chemotherapy with S-1 in CY1, a positive ascites cytology in the absence of distant metastasis. Patients who met the abovementioned criteria were divided into low visceral, high visceral, low subcutaneous, and high subcutaneous fat groups. This study was conducted according to the guidelines laid down in the Declaration of Helsinki and all procedures involving human subjects/patients were approved by the Institutional Ethical Review Committee of Ishikawa Prefectural Central Hospital (authorization number: 1747). Written informed consent was obtained from all subjects/patients.

### 2.2. Postoperative Treatment

In this study, the postoperative adjuvant chemotherapy regimen was S-1, started at 80–120 mg/m^2^/day, and reduced according to guidelines if side effects were observed. Postoperative chemotherapy with S-1 was continued for a maximum of 1 year, and no other treatment was administered until recurrence. Patients with recurrence received chemotherapy following the treatment guidelines.

### 2.3. Body Composition

We measured the preoperative visceral fat area and skeletal muscle mass on plain CT images using Ziostation graphical analysis software (ZIOSOFT, Tokyo, Japan). The visceral and subcutaneous fat masses were defined as tissues with a density from −160 to −70 Hounsfield units and were measured at the umbilical level. In addition, the skeletal muscle mass was defined as tissues with a density from −29 to 150 Hounsfield units and was measured at the level of the third lumbar vertebra. The visceral fat, subcutaneous fat, and skeletal muscle mass measured on a single CT image slice were divided by the patient’s height in meters squared to obtain the visceral fat mass index (VFI), subcutaneous fat mass index (SFI), and skeletal muscle mass index (SMI), respectively [16].

The cut-off values for VFI, SFI, and SMI were estimated separately for men and women based on the median of each group. The cut-off values for VFI, SFI, and SMI were 35.42 cm^2^/m^2^ for men and 26.81 cm^2^/m^2^ for women, 33.90 cm^2^/m^2^ for men and 41.70 cm^2^/m^2^ for women, and 41.87 cm^2^/m^2^ for men and 34.04 cm^2^/m^2^ for women, respectively. Patients whose VFI, SFI, and SMI values were above or below the cut off-values were categorized as having high or low values, respectively.

### 2.4. Outcomes and Analyses

The primary outcome was overall survival (OS) defined as the period between surgery and death. OS was compared between the two groups defined by VFI and SFI in all patients and by sex. We also compared OS in all patients stratified into four groups based on VFI and SFI. Finally, prognostic factors by sex were investigated using multivariate analysis.

We used the Mann–Whitney U test for continuous variables, the Chi-square test or Fisher’s exact test for categorical variables, and the log-rank test for Kaplan–Meier survival analysis for OS. We used the Cox proportional hazards regression for univariate analysis to identify prognostic factors for OS with *p* values < 0.05, of which a multivariate analysis was performed to calculate hazard ratios (HRs). We performed all statistical analyses using EZR software (Saitama Medical Center, Jichi Medical University, Saitama, Japan), which is based on R software (The R Foundation for Statistical Computing, Vienna, Austria) and R Commander. Statistical significance was set at *p* < 0.05.

## 3. Results

### 3.1. Patient Characteristics

The characteristics of the patients are shown in Table 1. A total of 485 patients (323 [66.6%] men, 162 [33.4%] women) were included. The men had a higher mean BMI (*p* < 0.001), a higher percentage of lymph node metastases (*p* = 0.023), a higher percentage of differentiated cancers (*p* = 0.002), a higher incidence of COPD (*p* = 0.032) and CHF (*p* = 0.003), higher VFI (*p* = 0.010), lower SFI (*p* < 0.001), higher SMI (*p* < 0.001), a higher incidence of Clavien–Dindo grade two or higher complications (*p* = 0.019), a higher incidence of Clavien–Dindo grade three or higher complications (*p* = 0.007), and a higher incidence of infectious complications (*p* = 0.011).

### 3.2. OS by VFI

**Figure 1:**
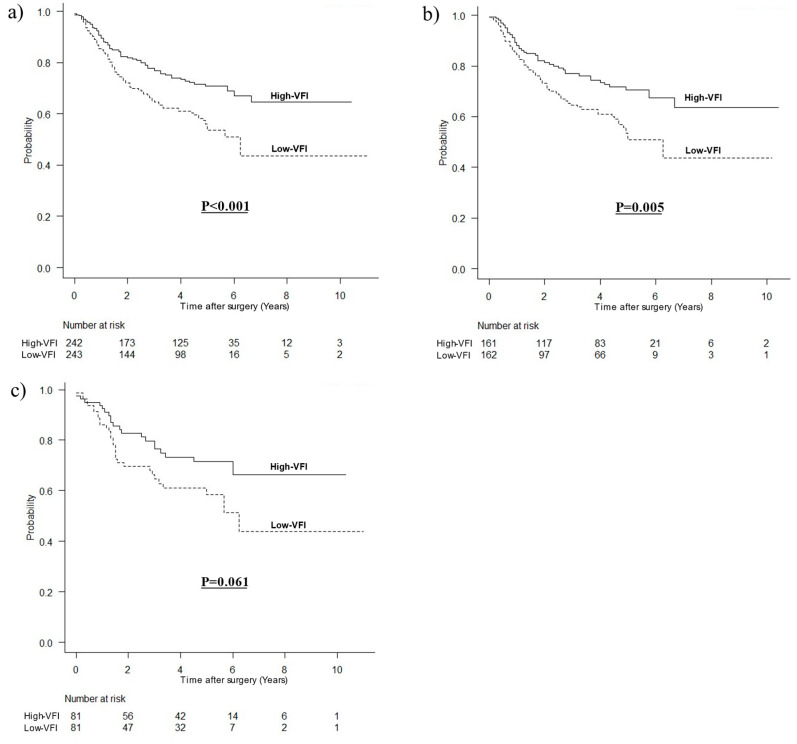
Kaplan–Meier survival curves for OS according to visceral fat mass. (**a**) OS for all patients (*p* < 0.001), (**b**) OS for men (*p* = 0.005), (**c**) OS for women (*p* = 0.061).

The median follow-up period was 41 (interquartile range, 16–60) months. The OS curve divided by visceral fat mass is shown in Figure 1. In all patients, the OS was significantly worse in the low VFI group (HR, 1.721; 95% confidence interval [CI], 1.255–2.360; *p* < 0.001). In men, the mean OS was significantly worse in the low VFI group (HR, 1.735; 95% CI, 1.182–2.547; *p* = 0.005). In women, the OS was worse in the low VFI group (HR, 1.700; 95% CI, 0.975–2.963; *p* = 0.061).

### 3.3. OS by SFI

**Figure 2:**
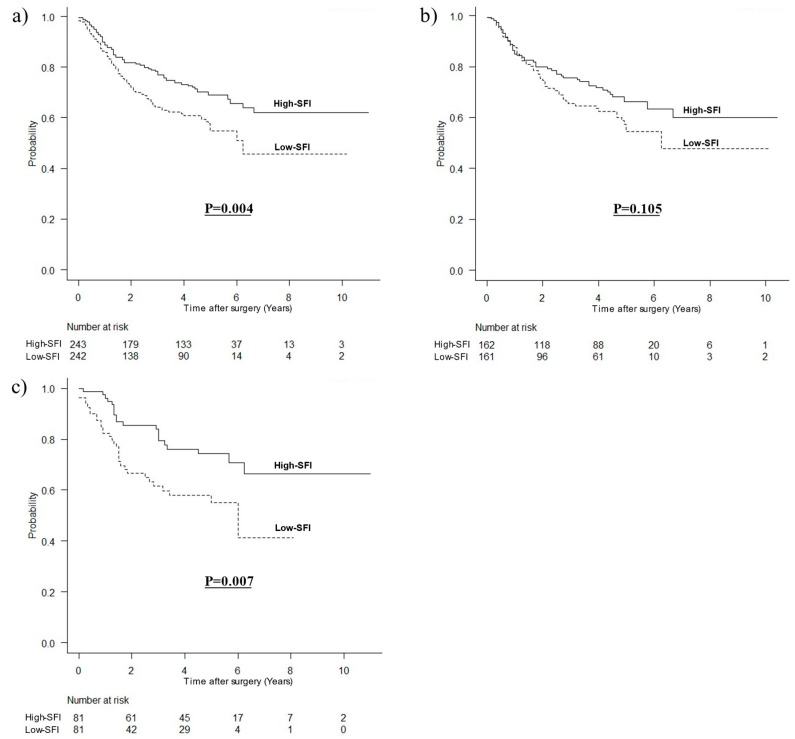
Kaplan–Meier survival curves for OS according to subcutaneous fat mass. (**a**) OS for all patients (*p* = 0.004), (**b**) OS for men (*p* = 0.105), (**c**) OS for women (*p* = 0.007).

The OS curve divided by subcutaneous fat mass is shown in Figure 2. In all patients, the OS was significantly worse in the low SFI group (HR, 1.584; 95% CI, 1.158–2.166; *p* = 0.004). In men, the OS was worse in the low SFI group (HR, 1.368; 95% CI, 0.937–1.996; *p* = 0.105). In women, the OS was significantly worse in the low SFI group (HR, 2.190; 95% CI, 1.240–3.868; *p* = 0.007).

### 3.4. OS Stratified by VFI and SFI

**Figure 3:**
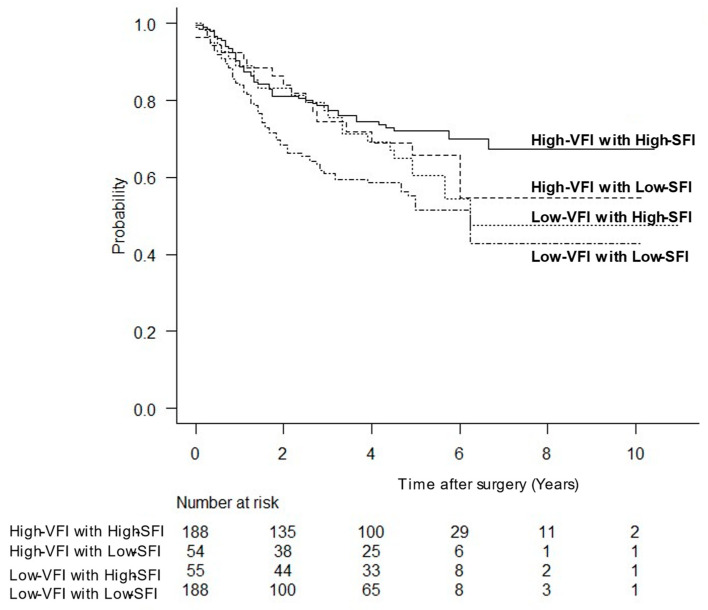
Kaplan–Meier survival curves for overall survival stratified by visceral fat and subcutaneous fat masses.

The OS rates stratified by VFI and SFI are shown in Figure 3. Patients with a low VFI and a low SFI had the worst survival rates, while those with a high VFI and a high SFI had the best survival rates.

### 3.5. Prognostic Factors for OS in Men

The results of the prognostic factor analysis for OS in men are shown in Table 2. In the univariate analysis, age > 70 years (*p* < 0.001), total gastrectomy (*p* < 0.001), open surgery (*p* < 0.001), serosal invasion (*p* < 0.001), lymph node metastasis (*p* < 0.001), a low SMI (*p* < 0.001), and a low VFI (*p* = 0.005) were statistically significant. The multivariate analysis showed that age > 70 years (HR, 2.229; 95% CI, 1.495–3.322; *p* < 0.001), total gastrectomy (HR, 1.616; 95% CI, 1.096–2.382; *p* = 0.015), open surgery (HR, 1.930; 95% CI, 1.258–2.962; *p* = 0.003), serosal invasion (HR, 1.973; 95% CI, 1.289–3.020; *p* = 0.002), N3 lymph node metastasis (HR, 2.143; 95% CI, 1.394–3.294; *p* < 0.001), and a low VFI (HR, 1.506; 95% CI, 1.019–2.226; *p* = 0.040) were all significant independent prognostic factors for OS.

### 3.6. Prognostic Factors for OS in Women

The results of the prognostic factor analysis for OS in women are shown in Table 3. In the univariate analysis, age > 70 years (*p* = 0.005), open surgery (*p* < 0.001), D2 lymph node dissection (*p* = 0.043), serosal invasion (*p* = 0.001), N3 lymph node metastasis (*p* < 0.001), and a low SFI (*p* = 0.007) were all statistically significant. The multivariate analysis showed that age > 70 years (HR, 1.926; 95% CI, 1.092–3.397; *p* = 0.024), open surgery (HR, 2.088; 95% CI, 1.107–3.938; *p* = 0.023), D2 lymph node dissection (HR, 0.359; 95% CI, 0.196–0.658; *p* < 0.001), N3 lymph node metastasis (HR, 3.412; 95% CI, 1.845–6.308; *p* < 0.001), and a low SFI (HR, 2.016; 95% CI, 1.115–3.643; *p* = 0.020) were all significant independent prognostic factors.

## 4. Discussion

This study showed that the prognosis of patients with advanced gastric cancer was better among men with a high visceral fat mass and women with a high subcutaneous fat mass. VFI and SFI were positively correlated, but when all patients were stratified by visceral fat mass and subcutaneous fat mass, patients with a high VFI and a high SFI had the best prognosis, while those with a low VFI and a low SFI had the worst prognosis. When stratified by skeletal muscle mass, a low SMI was associated with a poorer OS in men but not in women. This is the first study to show the impact of sex-based differences in body composition on long-term prognosis in patients with advanced gastric cancer after gastrectomy.

The multivariate analysis showed that a low VFI was a poor prognostic factor in men, whereas a low SFI was not. There was a positive correlation between VFI and SFI, but one was not included as a factor in the multivariate analysis, so it did not affect the results of the analysis. In the stratified OS comparison, a high VFI and a high SFI had the best prognosis, while a low VFI and a low SFI had the worst prognosis, suggesting that each factor may have a different effect. The reason for the better prognosis in patients with a higher fat content is thought to be the body weight loss associated with the decrease in gastric volume after gastrectomy. Reportedly, the greater the rate of body weight loss, the worse the long-term prognosis [17,18,19,20]. Body weight loss often lasts for 6 months, and skeletal muscle mass decreases mainly in the acute phase after surgery, while fat mass decreases thereafter [21,22,23,24,25]. A preoperative high fat mass reflects nutrient accumulation, and differences in fat accumulation in men and women may have led to the different impacts on prognosis. Harada et al. reported that a low visceral fat mass was associated with a poor prognosis in patients with upper gastrointestinal cancers [26]. They pointed out that low visceral fat mass is a state of undernutrition, whereas accumulated fat mass is a source of energy when one’s energy balance is negative. Park et al. reported that a marked decrease in visceral fat, subcutaneous fat, or skeletal muscle mass after gastrectomy was associated with a poor prognosis [27]. On the other hand, Dong et al. reported that excessive visceral fat mass is associated with poor prognosis in patients with BMI > 25 kg/m^2^ [28]. Based on these results, we should recognize that a low visceral fat mass is associated with a poor prognosis, rather than an excessive visceral fat mass being good. Therefore, fat mass was protective in this study, which included many patients with BMI < 25 kg/m^2^, and a high visceral fat mass in men and a high subcutaneous fat mass in women may have been a good prognostic factor.

In univariate analysis, skeletal muscle mass correlated with prognosis in men but not in women. The relationship between muscle mass and prognosis in patients with gastric cancer has not been examined by gender. It has been reported that changes in sex hormones caused by aging and disease are a major factor in muscle wasting [29,30,31]. In men, the decline in testosterone is particularly important, and muscle mass decreases with age [29,30]. It has been suggested that progesterone and estrogen may regulate muscle mass in women [31]. Men are characterized by a greater but more easily decreasing muscle mass than women [32]. Since these sex hormones are involved in the maintenance of muscle mass, they may also be involved in the prognosis of cancer patients.

The present study examined the validity of these methods for assessing body composition. While BMI is a convenient indicator of obesity, it alone cannot be used to determine the breakdown of skeletal muscle mass, visceral fat mass, and subcutaneous fat mass. CT is the gold standard for measuring visceral adiposity [33], and increasing recent reports detail its use to measure body composition. Kobayashi et al. showed that visceral fat area in a single slice measured at the umbilical level correlated strongly with the visceral fat mass of the entire body [34]. Therefore, it is highly likely that the single-slice measurements of fat mass in this study reflect the accumulation of fat mass throughout the body. In this study, we also used height-corrected indices to calculate the cut-off values for fat mass. The cut-off values were defined as median values, but their validity is unknown and future studies are needed. In recent years, body composition assessments have become more common, and it is now possible to assess body composition using both medical measuring devices and home measuring devices. Therefore, the need for preoperative assessments of body composition as a prognostic factor is expected to increase.

The limitations of this study are as follows: First, it was a single-center retrospective cohort study. Further prospective multicenter studies are needed. Second, the differences in body size among various races must be taken into consideration. As such, Asians tend to have a lower BMI and are less likely to be obese than Europeans, which may have affected our results. Further research outside of Asia is needed. Third, the cut-off values require validation in additional multicenter cohort studies. However, this is the first report to show the crucial impact of sex-specific preoperative fat mass assessments on postoperative long-term prognosis in patients with advanced gastric cancer who exhibit postoperative body weight loss. Our findings imply that there is a high need for postoperative nutritional support in the low VFI group for men and the low SFI group for women. In the future, we will investigate whether supportive therapies, including nutritional therapy, can contribute to a longer OS in such patients.

## 5. Conclusions

A low visceral fat mass in men and a low subcutaneous fat mass in women were independent poor prognostic factors after radical gastrectomy in patients with advanced gastric cancer. The group with low visceral and subcutaneous fat masses had the worst prognosis. These results suggest that differences in fat distribution between men and women may affect their long-term prognosis after gastrectomy.

## Figures and Tables

**Table 1:** Patient characteristics.

	All Patients (n = 485)	Men (n = 323)	Women (n = 162)	*p* Value
Age, mean ± SD	67.89 ± 11.1	67.94 ± 10.48	67.78 ± 12.35	0.886
Body mass index, mean ± SD	22.8 ± 3.52	23.28 ± 3.30	21.87 ± 3.77	<0.001
Surgical approach, Laparoscopic Open	248 (51.1%) 237 (48.9%)	158 (48.9%) 165 (51.1%)	90 (55.6%) 72 (44.4%)	0.178
Surgical procedure, Distal gastrectomy Proximal gastrectomy Total gastrectomy	265 (54.6%) 24 (4.9%) 196 (40.4%)	170 (52.6%) 15 (4.6%) 138 (42.7%)	95 (56.8%) 9 (5.6%) 58 (35.8%)	0.321
Lymph node dissection, D1+ D2	224 (46.2%) 261 (53.8%)	146 (45.2%) 177 (54.8%)	78 (48.1%) 84 (51.9%)	0.563
Pathological stage, I II III IV	83 (17.1%) 168 (34.6%) 180 (37.1%) 54 (11.1%)	59 (18.3%) 101 (31.3%) 129 (39.9%) 34 (10.5%)	24 (14.8%) 67 (41.4%) 51 (31.5%) 20 (12.3%)	0.060
Serosal invasion, Absent Present	350 (72.2%) 135 (27.8%)	240 (74.3%) 83 (25.7%)	110 (67.9%) 52 (32.1%)	0.162
Lymph node metastasis, Absent Present	130 (26.8%) 355 (73.2%)	76 (23.5%) 247 (76.5%)	54 (33.3%) 108 (66.7%)	0.023
Histological type, Differentiated Undifferentiated	207 (42.7%) 278 (57.3%)	154 (47.7%) 169 (52.3%)	53 (32.7%) 109 (67.3%)	0.002
Comorbidity, CKD COPD Diabetes CHF	88 (18.1%) 100 (20.6%) 91 (18.8%) 26 (5.4%)	64 (19.8%) 76 (23.5%) 68 (21.1%) 24 (7.4%)	24 (14.8%) 24 (14.8%) 23 (14.2%) 2 (1.2%)	0.212 0.032 0.084 0.003
VFI (cm^2^/m^2^), median (IQR) Low VFI	32.46 (16.69–51.02) 243 (50.1%)	35.42 (20.28–51.32) 162 (50.2%)	26.81 (11.59–46.22) 81 (50.0%)	0.010 1.000
SFI (cm^2^/m^2^), median (IQR) Low SFI	36.32 (21.70–53.83) 242 (49.9%)	33.90 (19.65–49.50) 161 (49.8%)	41.70 (26.19–71.43) 81 (50.0%)	<0.001 1.000
SMI (cm^2^/m^2^), median (IQR) Low SMI	39.08 (33.98–45.33) 242 (49.9%)	41.87 (37.33–47.77) 161 (49.8%)	34.04 (29.79–38.26) 81 (50.0%)	<0.001 1.000
Postoperative complication Clavien–Dindo grade ≥ 2 Clavien–Dindo grade ≥ 3 Infectious complications	105 (21.6%) 49 (10.1%) 66 (13.6%)	80 (24.8%) 41 (12.7%) 53 (16.4%)	25 (15.4%) 8 (4.9%) 13 (8.0%)	0.019 0.007 0.011

Chronic heart failure; chronic kidney disease; chronic obstructive pulmonary disease; interquartile range; standard deviation; subcutaneous fat mass index; skeletal muscle mass index; visceral fat mass index.

**Table 2:** Results of univariate and multivariate analyses of prognostic factors for overall survival in men.

Variables	Univariate Analysis			Multivariate Analysis		
	HR	95% CI	*p* Value	HR	95% CI	*p* Value
Age (years) <70 ≥70	1 2.213	1.515–3.231	<0.001	1 2.229	1.495–3.322	<0.001
Surgical procedure, Distal gastrectomy Total gastrectomy	1 1.915	1.315–2.788	<0.001	1 1.616	1.096–2.382	0.015
Surgical approach, Laparoscopic Open	1 2.839	1.882–4.284	<0.001	1 1.930	1.258–2.962	0.003
Lymph nodes dissection, D1+ D2	1 0.716	0.493–1.039	0.079			
Serosal invasion, Absent Present	1 3.031	2.075–4.428	<0.001	1 1.973	1.289–3.020	0.002
Lymph node metastasis, Absent Present N2 N3	1 3.532 3.005 3.489	1.893–6.590 2.004–4.506 2.391–5.091	<0.001 <0.001 <0.001	1 2.143	1.394–3.294	<0.001
Adjuvant chemotherapy, Absent Present	1 1.105	0.732–1.666	0.635			
Histological type, Differentiated Undifferentiated	1 1.443	0.988–2.110	0.058			
Chronic kidney disease, Absent Present	1 1.037	0.639–1.684	0.882			
Diabetes, Absent Present	1 1.423	0.916–2.211	0.116			
COPD, Absent Present	1 1.501	0.991–2.275	0.055			
Chronic heart failure, Absent Present	1 0.843	0.392–1.813	0.662			
SMI (cm^2^/m^2^), High-SMI Low-SMI	1 2.226	1.506–3.290	<0.001	1 1.416	0.941–2.131	0.096
VFI (cm^2^/m^2^), High-VFI Low-VFI	1 1.735	1.182–2.547	0.005	1 1.506	1.019–2.226	0.040
SFI (cm^2^/m^2^), High-SFI Low-SFI	1 1.368	0.937–1.996	0.105			
Infectious complication, Absent Present	1 1.246	0.760–2.043	0.384			
Postoperative complication, Absent Clavien–Dindo ≥ 2 Clavien–Dindo ≥ 3	1 0.994 1.563	0.640–1.542 0.944–2.590	0.977 0.083			

Chronic obstructive pulmonary disease; confidence interval; hazard ratio; subcutaneous fat mass index; skeletal muscle mass index; visceral fat mass index.

**Table 3:** Results of univariate and multivariate analyses of prognostic factors for overall survival in women.

Variables	Univariate Analysis			Multivariate Analysis		
	HR	95% CI	*p* Value	HR	95% CI	*p* Value
Age (years) <70 ≥70	1 2.227	1.273–3.898	0.005	1 1.926	1.092–3.397	0.024
Surgical procedure, Distal gastrectomy Total gastrectomy	1 1.618	0.943–2.775	0.081			
Surgical approach, Laparoscopic surgery Open surgery	1 2.863	1.620–5.059	<0.001	1 2.088	1.107–3.938	0.023
Lymph nodes dissection, D1+ D2	1 0.569	0.329–0.983	0.043	1 0.359	0.196–0.658	<0.001
Serosal invasion, Absent Present	1 2.464	1.437–4.226	0.001	1 1.686	0.935–3.042	0.083
Lymph node metastasis, Absent Present N2 N3	1 1.654 1.963 3.408	0.895–3.055 1.126–3.424 1.956–5.940	0.108 0.017 <0.001	1 3.412	1.845–6.308	<0.001
Adjuvant chemotherapy, Absent Present	1 0.623	0.363–1.068	0.085			
Histological type, Differentiated Undifferentiated	1 0.656	0.378–1.139	0.134			
Chronic kidney disease, Absent Present	1 1.183	0.577–2.426	0.646			
Diabetes, Absent Present	1 0.795	0.358–1.763	0.572			
COPD, Absent Present	1 0.694	0.297–1.625	0.400			
SMI (cm^2^/m^2^),High-SMI Low-SMI	1 1.207	0.699–2.082	0.500			
VFI (cm^2^/m^2^), High-VFI Low-VFI	1 1.700	0.975–2.963	0.061			
SFI (cm^2^/m^2^), High-SFI Low-SFI	1 2.190	1.240–3.868	0.007	1 2.016	1.115–3.643	0.020
Infectious complication, Absent Present	1 0.943	0.339–2.626	0.911			
Postoperative complication, Absent Clavien–Dindo ≥ 2 Clavien–Dindo ≥ 3	1 1.022 1.968	0.459–2.275 0.702–5.521	0.957 0.198			

Chronic obstructive pulmonary disease; confidence interval; hazard ratio; subcutaneous fat mass index; skeletal muscle mass index; visceral fat mass index.

## Data Availability

The datasets generated and/or analyzed during the current study are available upon reasonable request from the corresponding author.
